# To expand coverage, or increase frequency: Quantifying the tradeoffs between equity and efficiency facing cervical cancer screening programs in low‐resource settings

**DOI:** 10.1002/ijc.30551

**Published:** 2017-01-27

**Authors:** Nicole G. Campos, Vivien Tsu, Jose Jeronimo, Mercy Mvundura, Kyueun Lee, Jane J. Kim

**Affiliations:** ^1^ Harvard T.H. Chan School of Public Health Center for Health Decision Science 718 Huntington Avenue Boston MA; ^2^ PATH, Reproductive Health Program P.O. Box 900922 Seattle WA; ^3^ Devices and Tools Program PATH P.O. Box 900922 Seattle WA; ^4^ Department of Health Research and Policy Stanford University Stanford CA

**Keywords:** human papillomavirus, Uganda, cervical cancer, cancer screening, HPV DNA test, economic evaluation, health disparities

## Abstract

Cervical cancer is a leading cause of cancer death worldwide, with 85% of the disease burden residing in less developed regions. To inform evidence‐based decision‐making as cervical cancer screening programs are planned, implemented, and scaled in low‐ and middle‐income countries, we used cost and test performance data from the START‐UP demonstration project in Uganda and a microsimulation model of HPV infection and cervical carcinogenesis to quantify the health benefits, distributional equity, cost‐effectiveness, and financial impact of either (1) improving access to cervical cancer screening or (2) increasing the number of lifetime screening opportunities for women who already have access. We found that when baseline screening coverage was low (*i.e*., 30%), expanding coverage of screening once in a lifetime to 50% can yield comparable reductions in cancer risk to screening two or three times in a lifetime at 30% coverage, lead to greater reductions in health disparities, and cost 150 international dollars (I$) per year of life saved (YLS). At higher baseline screening coverage levels (*i.e*., 70%), screening three times in a lifetime yielded greater health benefits than expanding screening once in a lifetime to 90% coverage, and would have a cost‐effectiveness ratio (I$590 per YLS) below Uganda's per capita GDP. Given very low baseline coverage at present, we conclude that a policy focus on increasing access for previously unscreened women appears to be more compatible with improving both equity and efficiency than a focus on increasing frequency for a small subset of women.

## Abstract 2

**What's new?**

Most cervical cancer cases and deaths occur in less‐developed countries, where resource constraints challenge the planning and implementation of screening programs. The present report examines tradeoffs between equity and efficiency in cervical cancer screening approaches specifically in Uganda, where current baseline screening coverage is low. Analyses indicate that the expansion of access to once‐in‐a lifetime cervical cancer screening in areas with initially low baseline coverage is likely to yield greater benefits for health, distributional equity and cost‐effectiveness than increasing the number of screening opportunities per woman in low‐resource settings. Improving access for previously unscreened women should be a priority in such areas.

Cervical cancer is a leading cause of cancer death worldwide, with 85% of the disease burden residing in less developed regions.^1^ Yet cervical cancer is preventable. The opportunity for primary prevention exists with the availability of two prophylactic vaccines efficacious against HPV types 16 and 18, which cause approximately 70% of cervical cancers, and a recently approved 9‐valent vaccine against five additional oncogenic HPV types.^2^, ^3^, ^4^, ^5^ The HPV 16/18 vaccines are rolling out with support from Gavi, the Vaccine Alliance, in at least 20 low‐ and lower middle‐income countries.^6^ Despite the promise of vaccines, scale‐up of programs targeting young adolescent girls will take time. For two to three generations of women beyond the target age of vaccination, cervical cancer screening to detect and treat precancerous lesions remains the only form of prevention.

Where resources are available, the World Health Organization (WHO) recommends a “screen‐and‐treat” strategy for women aged 30 to 49 years, screening with HPV testing and treating eligible HPV‐positive women with timely cryotherapy.^7^ Where resources for organized screening with HPV testing are insufficient, the WHO recommends visual inspection with acetic acid (VIA), a low‐cost screening test that can provide immediate results. However, VIA is considerably less sensitive than HPV testing and necessitates stringent quality control measures and provider training.^7^

Implementing HPV‐based “screen‐and‐treat” programs in low‐resource settings is logistically difficult because of constraints associated with laboratory processing and availability of cryotherapy. The *care*HPV test (Qiagen, Gaithersburg, MD, USA) has minimal laboratory requirements, but the testing system is designed to be run in batch mode,^8^ likely necessitating transport of samples to a centralized location. The laboratory processing time of approximately four hours hinders same‐day results and treatment for HPV‐positive women. Furthermore, cryotherapy is not available at all primary health facilities because of the high cost of equipment and the need for consistent resupply of gas.^9^ As a result, a “screen‐and‐treat” approach with HPV testing may be more favorable for women in urban areas, where greater access to laboratory and treatment facilities may lead to more timely follow‐up and management of screen‐positive women. However, pilot studies demonstrate that HPV self‐collection facilitated by community health workers may increase screening uptake among underserved women,^10^, ^11^, ^12^, ^13^ thereby expanding access to care if HPV‐positive women can be successfully navigated to a treatment facility.

As cervical cancer screening programs are planned, implemented, and scaled in low‐ and middle‐income countries, decision‐makers will face tradeoffs as they seek to maximize population health or equitably distribute health benefits given resource constraints. One tradeoff is whether to concentrate resources on (1) expanding coverage of a single lifetime screen in the general population of screening‐eligible women, or (2) offering multiple screening opportunities (*i.e*., two or three screenings in a lifetime) to women who already have access. The health outcomes and cost‐effectiveness of a screening program may depend on whether the program prioritizes greater access versus higher frequency of screening. To inform evidence‐based decision‐making, we integrated cost and test performance data from the START‐UP demonstration project in Uganda into a microsimulation model of HPV infection and cervical carcinogenesis. Our objective was to quantify the health benefits, distributional equity, cost‐effectiveness and financial impact of expanding screening coverage to more women versus increasing screening frequency for a select population.

## Methods

### Analytic overview

We used an existing individual‐based Monte Carlo simulation model of the natural history of HPV and cervical cancer to estimate lifetime health and economic outcomes associated with screening with HPV DNA testing.^14^, ^15^ We compared screening two or three times in a lifetime at baseline screening coverage — which we defined as existing coverage with once in a lifetime screening, ranging from 30% to 80%— against screening once in a woman's lifetime as screening coverage varied from baseline to 90%. The model was calibrated to epidemiologic data from Uganda.^14^ Test performance and cost data were obtained from the START‐UP demonstration project in Kampala, Uganda.^16^, ^17^ Model outcomes included lifetime cervical cancer risk, total lifetime costs and life expectancy. We defined a health disparity as the difference in life expectancy between women with and without access to screening, and distributional equity as the difference between life expectancy in women with access to screening and population average life expectancy (which depended upon screening coverage). We calculated incremental cost‐effectiveness ratios (ICERs), defined as the additional cost of a particular strategy divided by its additional health benefit, compared with the next most costly strategy after eliminating strategies that are dominated (defined as more costly and less effective, or having higher ICERs than more effective options). While there is no universal criterion that defines a threshold cost‐effectiveness ratio, we followed the convention that an intervention with an ICER less than Uganda's per capita gross domestic product (GDP) would be “very cost‐effective” and less than three times per capita GDP would be “cost‐effective”.^18^ Consistent with guidelines for cost‐effectiveness analysis, we adopted a societal perspective, including costs irrespective of the payer; we discounted future costs and life‐years at a rate of 3% per year to account for time preferences.^19^, ^20^, ^21^

### Mathematical simulation model

The natural history model of cervical carcinogenesis and a description of the model parameterization process have been previously published.^14^, ^15^, ^22^, ^1^

An individual woman is represented as a sequence of monthly transitions between mutually exclusive health states, including type‐specific HPV infection status, grade of precancer (*i.e*., cervical intraepithelial neoplasia [CIN] grade 2 or 3), and stage of invasive cancer. Individual girls enter the model at age 9 with a healthy cervix, and are simulated until death. Transition probabilities may vary by age, HPV type, duration of infection or precancerous lesion status, and prior HPV infection. Cancer detection can occur through symptoms or *via* screening. Each month, death can occur from non‐cervical causes or from cervical cancer after its onset. The model tracks disease progression and regression, clinical events, and economic outcomes over the lifetime of each individual woman, which are then aggregated for analysis.

For natural history transitions, we estimated baseline “prior” input parameter values and set plausible ranges using epidemiologic data.^14^, ^15^, ^23^, ^24^, ^25^, ^26^ We conducted repeated model simulations in the absence of any intervention, in which a single random value for each uncertain parameter was selected from the plausible range, creating a unique natural history input parameter set. We then computed a goodness‐of‐fit score by summing the log‐likelihood of model‐projected outcomes for each unique parameter set to represent the quality of fit to epidemiologic data from Uganda (*i.e*., calibration targets). We selected the top 50 input parameter sets that produced a good fit to the epidemiologic data to use in analyses as a form of probabilistic sensitivity analysis.^15^, ^26^, ^27^ Model fit to empirical data on age‐specific prevalence of oncogenic HPV and age‐specific cancer incidence is displayed in the Supporting Information Appendix. We report results as the mean of outcomes across these top 50 parameter sets; incremental cost‐effectiveness ratios are reported as the ratio of the mean costs divided by the mean effects of one strategy versus another across sets.^28^

### Screening strategies

We assumed screening with HPV DNA testing took place either once in a woman's lifetime at age 30 years, twice in a lifetime at ages 30 and 40 years, or three times in a lifetime at ages 30, 40 and 50 years. We varied baseline screening coverage— which we defined as existing coverage with once in a lifetime screening, ranging from 30% to 80%— and compared health and cost outcomes as either (1) screening coverage associated with onetime screening increased from baseline to 90% in 10% increments, or (2) screening frequency increased to two or three times in a lifetime at the baseline screening coverage level (30% to 80%). For screening two or three times in a lifetime, we made the simplifying assumption that the same women received each screening, while the remaining proportion of the population was never screened. We assumed women were screened by a provider during an initial screening visit, and returned for a second visit to obtain results unless they were lost to follow‐up. If a woman screened positive and was eligible, most received same‐day cryotherapy at the results visit. Treatment protocols for women who were not eligible for immediate cryotherapy and management following treatment, were based on current practice in Uganda and are documented in the Supporting Information Appendix. Test performance, treatment, and compliance parameters are displayed in Table 1 **.** 16, 17, 22, 29, 30, 31, 32, 33, 34

**Table 1:** Baseline values for model variables^a^

Variable [Reference]	Value
Population coverage of screening program^b^	
Screening once in a lifetime	30%–90%
Screening two or three times in a lifetime	30%–80%
Loss to follow‐up, results visit^c^	15%
Test sensitivity/specificity for CIN2+, *care*HPV [ 16]^d^	89%/82%
Eligibility for cryotherapy [ 22]	
No lesion or CIN1	100%
CIN2	85%
CIN3	75%
Cancer	10%
Proportion of eligible women receiving immediate cryotherapy following *care*HPV results	80%
Loss to follow‐up, additional cryotherapy visit^c^	10%
Loss to follow‐up, colposcopy and treatment visits for women ineligible for cryotherapy^c^	15%
Test sensitivity/specificity for CIN1+, colposcopy^e^	95%/51%
Loss to follow‐up, treatment visit for women with CIN1+^c^	15%
Effectiveness of cryotherapy [ 22, 29, 30, 31]	92%
Effectiveness of cryotherapy/LEEP following colposcopy [ 22, 31]	96%
Direct medical costs [ 14, 16, 17]^f^	
*care*HPV (cervical specimen)^g^	8.78
Colposcopy^h^	7.08
Colposcopy and biopsy^h^	32.90
Cryotherapy	13.49
LEEP	139.54
Direct non‐medical costs^f^	
Transportation (round‐trip, clinic) [ 22, 32, 33]	4.46
Transportation (round‐trip, secondary facility) [ 22, 32, 33]	10.87
Women's time (per hour) [ 34]	0.68
Treatment of local cancer (FIGO stages 1a‐2a) [ 22, 32, 33]^f^, ^i^	888
Treatment of regional/distant cancer (FIGO stages ≥2b) [ 22, 32, 33]^f^, ^i^	1,176

a Abbreviations: CIN: cervical intraepithelial neoplasia; FIGO: International Federation of Gynecology and Obstetrics; LEEP: loop electrosurgical excision procedure. Further details on unit cost assumptions are available in the Supporting Information Appendix.

b Screening once in a lifetime occurs at age 30 years; twice in a lifetime at ages 30 and 40 years; and three times in a lifetime at ages 30, 40, and 50 years. For strategies involving screening two or three times in a lifetime, the proportion of population coverage applies to the same women for each screening; the remainder of the population is assumed to receive no screening.

c Loss to follow‐up is defined as the proportion of women who do not return for each subsequent clinical encounter, relative to the previous visit. Loss to follow‐up applies to the results visit following *care*HPV testing, the cryotherapy visit (only for women who do not receive immediate cryotherapy in the same visit as receipt of results), and the diagnostic confirmation visit and treatment visit for women who are ineligible for cryotherapy.

d Provider‐collection of cervical HPV specimens was assumed for the primary analysis, for all screening frequencies.

e Test performance characteristics of colposcopy in START‐UP were derived from the worst diagnosis of the local pathologist relative to the worst diagnosis by a quality control pathologist (gold standard); we applied the treatment threshold of CIN1+, although this was not the treatment threshold in START‐UP. To derive test performance of colposcopy, we excluded histological classifications that were inadequate or with a histological classification other than negative, CIN1, CIN2, CIN3 or cancer. Because CIN1 is not a true underlying health state in the model, performance of colposcopy in the model is based on the underlying health states of no lesion, HPV infection, CIN2 or CIN3. For a treatment threshold of CIN1, we weighted sensitivity of colposcopy for women with HPV based on the country‐specific prevalence of CIN1 among women with HPV infections in the START‐UP studies.

f All costs are in 2011 international dollars (I$). The location of service delivery for each procedure, as well as time spent traveling, waiting for, and receiving care by procedure and country, are presented in the Supporting Information Appendix. In the START‐UP study, procedures were performed at secondary or tertiary facilities, and costs may overestimate or underestimate costs at primary health facilities due to differences in volume of procedures and overhead costs.

g This includes the cost of the careHPV test, which was assumed to be I$5 (as a tradable good, this is equivalent to US$5).

h In the absence of data from actual practice in low‐resource settings, the proportion of colposcopies that were accompanied by a biopsy was drawn from START‐UP data (95.6% in Uganda).

i All cancer costs presented include the value of women's time spent pursuing care and transportation to health facilities.

### Cost data

Cost data have been published elsewhere and are summarized in Table 1.^14^, ^17^ Direct medical costs of screening, diagnosis, and treatment of precancerous lesions were drawn from the START‐UP study, and included staff time, clinical supplies, drugs, clinical equipment, laboratory staff time, laboratory supplies and laboratory equipment. We converted local currency units to 2011 international dollars (I$), a hypothetical currency that provides a means of translating and comparing costs among countries, taking into account differences in purchasing power. We assumed the *care*HPV test kit was a tradable good valued at US$5, including the test, sampling brush, and container; for tradable goods such as the *care*HPV test, one US dollar is equivalent to one I$.

Women's transportation costs and the cost of women's time spent traveling, waiting for, and receiving care were dependent upon the facility level and were derived from START‐UP data and the published literature, as previously described.^14^, ^16^, ^17^, ^22^, ^32^, ^33^ Costs associated with cancer care by stage included direct medical costs, women's time costs, and transportation costs. Further details are described in the Supporting Information Appendix.

### Calculation of incremental net monetary benefit

To measure the added value of shifting from screening once in a lifetime at the baseline coverage level to screening with either greater frequency or greater coverage, we calculated the incremental net monetary benefit (INMB) for 1) screening three times in a lifetime (at baseline coverage) and 2) screening once in a lifetime (at higher coverage), relative to once in a lifetime (at baseline coverage). The INMB translates the incremental health benefit (additional life‐years gained from shifting to a strategy with either greater frequency or greater coverage) into monetary terms for a specified cost‐effectiveness threshold. To calculate the INMB, the life‐years gained are multiplied by the threshold; then the incremental cost (the change in the expected lifetime cost per woman from shifting to a strategy with greater frequency or greater coverage) is subtracted. Thus, the INMB represents the maximum dollar amount per woman by which the cost of an intervention can be increased to achieve an improvement (in either frequency or coverage) while remaining “very cost‐effective” (*i.e*., having an ICER below Uganda's per capita GDP).^35^ The formula for the INMB is available in the Supporting Information Appendix.

### Financial impact analysis

To assess the financial impact of a screening program (from a payer perspective) given a specified coverage level and frequency of screening, we used the individual‐based simulation model to estimate the expected direct medical cost per woman of each screening strategy, including the costs of screening and any relevant diagnostic testing and treatment of precancer. The financial impact analysis did not consider cost offsets from future cancer cases prevented or women's time and transportation costs that were considered in the cost‐effectiveness analysis. We report the cost per 100,000 women for each coverage level and frequency in 2013 US$ instead of I$to provide a meaningful estimate to the international and donor communities.

## Results

### Health benefits

**Figure 1:**
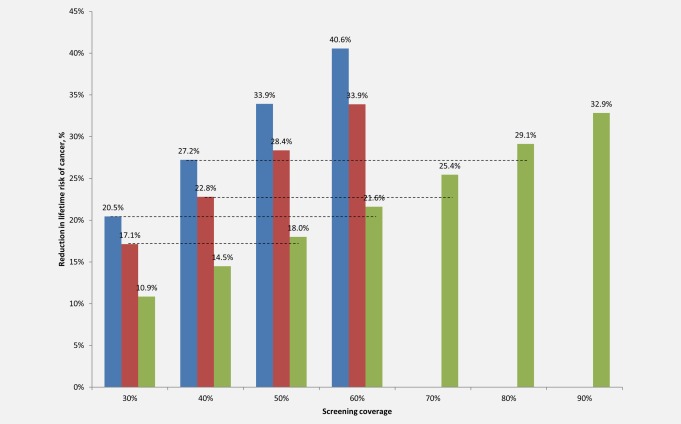
**Reduction in the lifetime risk of cervical cancer, by screening coverage level and frequency.** Reduction in lifetime risk of cervical cancer (*y*‐axis) is displayed by screening coverage level (*x*‐axis) for screening once, twice or three times in a lifetime with *care*HPV testing. Screening three times in a lifetime at ages 30, 40 and 50 years is displayed by the blue bars; screening twice in a lifetime at ages 30 and 40 years by the red bars; and screening once in a lifetime at age 30 years by the green bars. Dashed lines indicate the higher coverage level at which screening once in a lifetime (green bars) yields equal or greater reductions in cancer risk relative to baseline coverage levels of screening two (red bars) or three (blue bars) times in a lifetime. [Color figure can be viewed at wileyonlinelibrary.com]

Figure 1 shows the reduction in lifetime risk of cervical cancer, by level of coverage and screening frequency. At a baseline coverage level of 30%, screening once in a lifetime reduces cancer risk by 10.9%, screening twice in a lifetime by 17.1%, and screening three times in a lifetime by 20.5%. If coverage of once in a lifetime screening increases to 50%, cancer risk can be reduced by 18.0%, yielding greater health benefits than screening twice in a lifetime at baseline coverage. If coverage of once in a lifetime screening increases to 60%, cancer risk can be reduced by 21.6%, yielding greater health benefits than screening three times in a lifetime at baseline coverage.

As baseline coverage level rises, greater coverage increases are required for once in a lifetime screening to achieve comparable reductions in cancer risk relative to screening two or three times in a lifetime at baseline coverage. At a baseline coverage level of 40%, screening once, twice or three times in a lifetime reduces cancer risk by 14.5%, 22.8% or 27.2%, respectively. Expanding coverage of once in a lifetime screening to 70% of the target population reduces cancer risk by 25.4%, exceeding the health benefits associated with screening twice in a lifetime at baseline coverage. Expanding coverage of onetime screening to 80% reduces cancer risk by 29.1%, exceeding the health benefits associated with screening three times in a lifetime at baseline coverage.

At a baseline coverage level of 50%, screening once, twice or three times in a lifetime reduces cancer risk by 18.0%, 28.4% and 33.9%, respectively. Expanding coverage of once in a lifetime screening to 80% reduces cancer risk by 29.1%, and increasing coverage to 90% reduces cancer risk by 32.9%, exceeding the health benefits of screening twice in a lifetime at baseline coverage but yielding slightly lower cancer risk reductions than screening three times in a lifetime at baseline coverage. As the baseline coverage level reaches 60%, screening two or three times in a lifetime yields greater risk reduction than screening once in a lifetime at 90% coverage. Cancer risk reductions for baseline coverage levels of 70% and 80% are shown in the Supporting Information Appendix.

**Figure 2:**
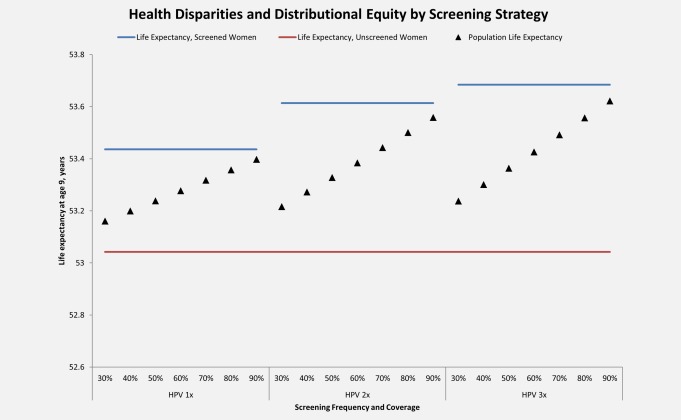
**Health disparities and distributional equity, by screening coverage level and frequency.** Life expectancy at age 9 (*y*‐axis) is displayed for each screening coverage and frequency considered. Life expectancy for unscreened women is represented by the red lines, life expectancy for screened women (100% coverage) is represented by the blue lines, and average female life expectancy for the general population with a specified screening coverage level and frequency is represented by the black triangles. 1x: screening once in a lifetime at age 30 years; 2x: screening twice in a lifetime at ages 30 and 40 years; 3x: screening three times in a lifetime at ages 30, 40, and 50 years. [Color figure can be viewed at wileyonlinelibrary.com]

Figure 2 displays the tradeoff between expanding coverage and increasing screening frequency in terms of health disparities in life expectancy. Screening once in a lifetime in Uganda can raise the average female (undiscounted) life expectancy at age 9 from 53.04 years (with no screening) to 53.44 years (with 100% coverage). Each additional screening in a woman's lifetime increases life expectancy, but to a lesser degree than the initial screen; screening twice or three times in a lifetime raises the life expectancy for screened women to 53.61 or 53.68 years, respectively. The disparity between screened and unscreened women thus increases as a greater number of screenings are offered to women already with screening access. For each 10% increase in coverage for once in a lifetime screening, the population average life expectancy increases by approximately 0.039 years, improving the distributional equity (*i.e*., the difference between the life expectancy for women with access to screening and the population average life expectancy). While each 10% increase in coverage for screening two or three times in a lifetime screening yields slightly greater increases in population average life expectancy (with average increases of approximately 0.057 years and 0.064 years, respectively) than comparable coverage gains with screening once in a lifetime, improvements in distributional equity are more modest. For instance, assuming baseline coverage of 60%, the life expectancy associated with screening twice in a lifetime is similar to the life expectancy associated with screening once in a lifetime with coverage at 90% (approximately 53.39 years), but the difference between the population average life expectancy and the life expectancy of screened women is 0.23 years when 60% of women have access to screening twice in a lifetime, and only 0.039 years when 90% women have access to screening once in a lifetime.

### Cost‐effectiveness analysis

**Figure 3:**
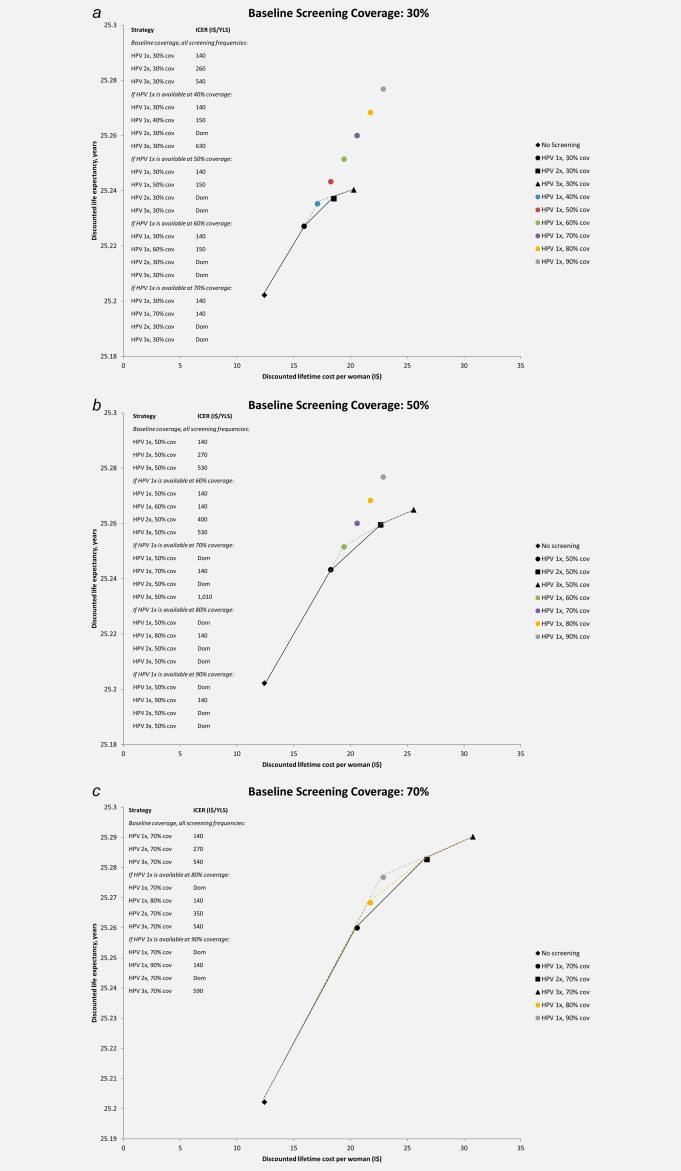
**Cost‐effectiveness of screening for cervical cancer.** The discounted lifetime costs (in 2011 international dollars) and life expectancy associated with selected screening coverage levels and frequency are shown for baseline coverage levels of (*a*) 30%; (*b*) 50%; and (*c*) 70%. Black markers represent the discounted costs and life expectancy for no screening (diamond), screening once in a lifetime (circle), twice in a lifetime (square), or three times in a lifetime (triangle) at the specified baseline coverage level. The cost‐effectiveness associated with a change from one strategy to a more costly alternative is represented by the difference in cost divided by the difference in life expectancy associated with the two strategies. Strategies that lie on the efficiency curve dominate those to the right of the curve because they are more effective and either cost less or have a more attractive cost‐effectiveness ratio than less effective options. An incremental cost‐effectiveness ratio is shown for each non‐dominated strategy and is the reciprocal of the slope of the line connecting the two screening strategies under comparison. This slope is steeper when the incremental gain in life expectancy per international dollar is greater. The black line represents the efficiency frontier when screening once, twice, or three times in a lifetime is available at baseline coverage levels only. In panel (*a*), the blue dashed line represents the efficiency frontier when once in a lifetime screening is also available at 40% coverage; when once in a lifetime screening is also available at coverage levels of 50% or higher, screening once in a lifetime is more effective and less costly than screening two or three times in a lifetime at baseline coverage (efficiency frontiers not shown). In panel (*b*), the green dashed line indicates the efficiency frontier when once in a lifetime screening is also available at 60% coverage; when once in a lifetime screening is also available at coverage levels of 70% or higher, screening once in a lifetime is more effective and less costly than screening twice in a lifetime (efficiency frontiers not shown). In panel (*c*), the yellow and gray dashed lines indicate the efficiency frontiers when once in a lifetime screening coverage is also available at 80% or 90% coverage, respectively. 1x: once in a lifetime screening at age 30 years; 2x: twice in a lifetime screening at ages 30 and 40 years; 3x: three times in a lifetime screening at ages 30, 40, and 50 years; cov: screening coverage level; dom: dominated strategy, defined as either more costly and less effective or having a higher incremental cost‐effectiveness ratio than a more effective strategy; I$: 2011 international dollars; ICER: incremental cost‐effectiveness ratio; YLS: year of life saved. Uganda GDP per capita: I$1,690. [Color figure can be viewed at wileyonlinelibrary.com]

The cost‐effectiveness of screening two or three times in a lifetime at baseline coverage relative to screening once in a lifetime at baseline coverage or greater is presented in Figure 3 for baseline coverage levels of 30%, 50% and 70% (see Supporting Information Appendix for all coverage levels). At a baseline screening coverage of 30%, screening once in a lifetime was associated with an ICER of I$140 per YLS, and screening twice in a lifetime with an ICER of I$260 per YLS; screening three times in a lifetime (I$540 per YLS) was the strategy that achieved the greatest life expectancy gains, and would be considered “very cost‐effective” with an ICER below Uganda's per capita GDP of I$1,690. When scenarios comparing improvements in coverage (*e.g*., once in a lifetime screening at 40% coverage) were compared against improvements in number of screens per lifetime (*e.g*., screening two or three times in a lifetime at baseline coverage of 30%), screening twice in a lifetime was no longer an efficient strategy. When once in a lifetime screening was available at 50% coverage or higher, it was less costly and more effective than screening two or three times in a lifetime at baseline coverage, with a stable ICER of I$140 to I$150 per YLS.

When baseline screening coverage was 50% for all screening frequencies, screening once, twice, or three times in a lifetime cost I$140 per YLS, I$270 per YLS, or I$530 per YLS, respectively. When once in a lifetime screening coverage was also assumed to be available at a higher coverage level of 60%, all strategies remained attractive, with ICERs below Uganda's per capita GDP. However, when once in a lifetime screening was assumed to be available at 70% coverage (I$140 per YLS), it was less costly and more effective than screening twice in a lifetime at baseline coverage levels; the ICER associated with screening three times in a lifetime at baseline coverage rose to I$1,010 per YLS. As coverage of once in a lifetime screening expanded to 80%, it was less costly and more effective than screening two or three times in a lifetime at baseline coverage, and its ICER remained stable at I$140 per YLS.

At a higher baseline screening coverage of 70%, screening once, twice or three times in a lifetime cost I$140 per YLS, I$270 per YLS, or I$540 per YLS, respectively. As screening coverage of once in a lifetime screening increased to 80%, screening two or three times in a lifetime remained attractive strategies. When coverage of once in a lifetime screening was assumed to be available at 90%, screening twice in a lifetime at baseline coverage was no longer cost‐effective. Screening three times in a lifetime remained the most effective strategy, and would be considered very cost‐effective with an ICER of I$590 per YLS.

In a sensitivity analysis, we assumed higher loss to follow‐up between visits; results are presented in the Supporting Information Appendix. Findings were qualitatively similar to the main analysis, although the value of screening more frequently in a woman's lifetime increased as women who had previously been lost to follow‐up had more opportunities for successful linkage to treatment. At low baseline coverage levels, once in a lifetime screening needed to reach higher coverage levels relative to the main analysis in order to achieve reductions in cancer risk that were comparable to screening two or three times in a lifetime.

**Figure 4:**
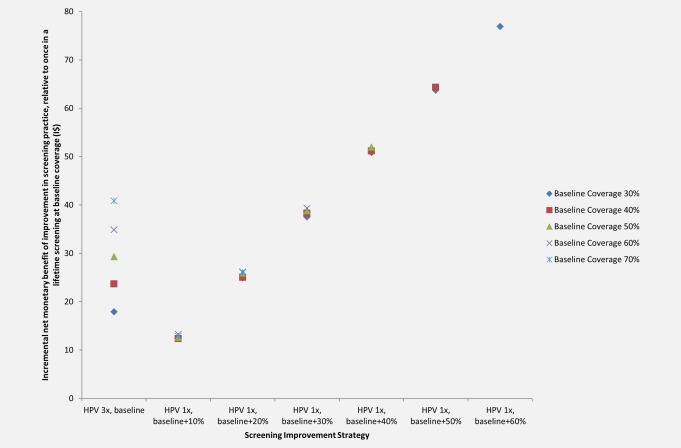
**Incremental net monetary benefit of increasing screening coverage versus increasing screening frequency.** The incremental net monetary benefit (INMB) of an improvement in screening practice relative to once in a lifetime screening at baseline coverage (*y*‐axis) is shown for each improvement (*x*‐axis), including increasing screening frequency to three times in a lifetime at baseline coverage or increasing screening coverage of once in a lifetime screening. INMB values are shown for baseline coverage levels of 30% (dark blue markers), 40% (red markers), 50% (green markers), 60% (purple markers), and 70% (turquoise markers). Uganda GDP per capita: I$1,690. 1x: once in a lifetime screening at age 30 years; 2x: twice in a lifetime screening at ages 30 and 40 years; 3x: three times in a lifetime screening at ages 30, 40, and 50 years; I$: 2011 international dollars. [Color figure can be viewed at wileyonlinelibrary.com]

The INMB values for shifting from once in a lifetime screening at baseline coverage to screening either three times in a lifetime (at baseline coverage) or once in a lifetime (at greater than baseline coverage) are presented in Figure 4. As baseline coverage levels increased, the INMB of shifting from screening once in a lifetime to three times in a lifetime increased, ranging from I$18 at 30% coverage to I$41 at 70% coverage.

The INMB of increasing coverage of screening once in a lifetime was dependent on the absolute coverage gain, and was fairly stable as baseline coverage increased. As baseline coverage varied from 30% to 70%, the INMB of an absolute gain in coverage of 10% ranged from I$12 to I$13; the INMB of a 20% gain in coverage ranged from I$25 to I$26; and the INMB of a 30% gain in coverage ranged from I$38 to I$39. While absolute coverage gains of 40% to 60% were only relevant when baseline screening coverage was 50% or less, the INMB values for these gains were high, ranging from approximately I$51 (for coverage gains of 40%) to I$77 (for coverage gains of 60%).

### Financial impact

**Figure 5:**
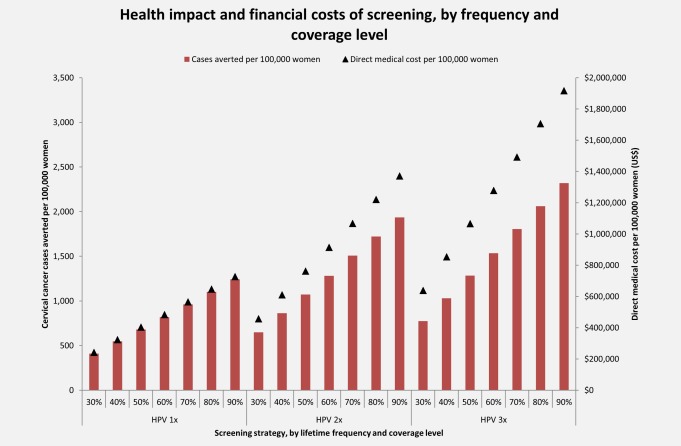
**Health impact and financial costs of screening, by screening coverage level and frequency.** The number of cervical cancer cases (red bars) averted per 100,000 women are shown on the primary *y*‐axis for each screening coverage level and frequency (*x*‐axis). The undiscounted direct medical costs (US$) (black triangles) per 100,000 women are displayed on the secondary y‐axis. 1x: once in a lifetime screening at age 30 years; 2x: twice in a lifetime screening at ages 30 and 40 years; 3x: three times in a lifetime screening at ages 30, 40, and 50 years; US$: 2013 US dollars. [Color figure can be viewed at wileyonlinelibrary.com]

The financial impact of a screening program for 100,000 women in a single birth cohort, juxtaposed to the projected number of cervical cancer cases averted, is presented in Figure 5 and the Supporting Information Appendix for each coverage level and frequency considered. For a specified payer's budget (in US$), decision‐makers can observe which strategy is projected to have the greatest health impact. For example, if a payer has up to US$5 per woman, screening once in a lifetime at 60% coverage will avert 817 cases of cervical cancer for a cost of US$480,000 per 100,000 women, while screening twice in a lifetime at 30% coverage will avert 648 cases for a cost of US$460,000 per 100,000 women. If a payer has up to US$9 per woman, screening once in a lifetime at 90% coverage will avert 1,242 cases of cervical cancer and will cost US$730,000 per 100,000 women. Alternatively, screening twice in a lifetime at 50% coverage will avert 1,073 cases at a cost of US$760,000 per 100,000 women, and screening three times in a lifetime at 40% coverage will avert 1,030 cases at a cost of US$850,000 per 100,000 women. In general, the health impact (in terms of cases averted) is greater for screening once in a lifetime at higher coverage rates than for programs with comparable costs that screen two or three times in a lifetime at lower coverage rates.

## Discussion

Our aim was to inform decision‐makers in low‐resource settings as they make programmatic choices to concentrate limited funds on (1) improving access to cervical cancer screening or (2) increasing the number of screening opportunities for women who already have access. We present both health and economic outcomes for a wide range of baseline coverage levels— which we define as existing coverage with once in a lifetime screening— as well as a range of coverage gains for once in a lifetime screening. We found that at low levels of baseline coverage (*i.e*., 30%) in Uganda, expanding coverage of screening once in a lifetime to 50% or more can yield comparable health benefits to screening two or three times in a lifetime at baseline coverage, and can be very cost‐effective. As the baseline coverage level rises to 50% and higher, extending the number of screens in women already with access becomes more attractive, and increasingly greater expansions in coverage for once in a lifetime screening are necessary to achieve comparable reductions in cancer risk. When baseline coverage reaches 70%, screening two or three times in a lifetime yielded greater health benefits than screening once in a lifetime at 90% coverage, and screening three times in a lifetime at baseline coverage of 70% was very cost‐effective. Thus, the relative efficiency of increasing screening coverage versus increasing frequency will depend on baseline coverage levels and achievable coverage gains. Importantly, we found that increasing coverage of once in a lifetime screening is more likely to reduce health disparities and improve distributional equity than screening a smaller proportion of the target population with greater frequency. For a specified payer's budget, more cases may be averted from achieving higher coverage with once in a lifetime screening than from increasing screening frequency.

Due to limited data from screening programs that are scaling up, it is not possible to estimate the differential programmatic costs or necessary capacity building of expanding coverage versus increasing screening frequency at this time. The costs and human resources required for expanding coverage to women in remote areas are potentially high. The programmatic costs and human resources required for screening women in urban areas with greater frequency might be relatively lower, if less outreach is required to bring women in for screening, fewer providers require training, and fewer pieces of equipment (including vehicles for sample transport) are needed for laboratory processing and treatment. In the absence of data on programmatic costs, we determined the maximum dollar amount per woman by which the cost of screening once in a lifetime (at baseline coverage) could be increased to achieve either higher coverage with once in a lifetime screening or greater screening frequency while remaining “very cost‐effective.” We calculated the INMB for each of these potential program improvements. At a low baseline coverage level of 30%, the INMB value for shifting from screening once in a lifetime to three times in a lifetime was I$18. This implies that the total per‐woman lifetime costs (including direct medical, non‐medical, women's time, and programmatic costs) of incorporating two additional screening episodes would need to be I$18 or less in order for screening three times in a lifetime to have an ICER below per capita GDP. As baseline coverage increased to 70%, the INMB for shifting from screening once in a lifetime to three times in a lifetime was much higher (I$41), reflecting the greater value of increasing screening frequency when most women already have access to screening at least once in a lifetime. The INMB values for increasing coverage of once in a lifetime screening steadily increased along with coverage gains, and indicate that total per‐woman lifetime costs could increase by I$12 to I$13 in order for a 10% absolute coverage gain to remain very cost‐effective, while costs could increase by I$38 to I$39 for a 30% coverage gain, regardless of baseline coverage. The presentation of INMB findings allows decision‐makers to assess the value of different screening program improvements, depending on baseline coverage and expected costs of either increasing frequency of screening or expanding coverage of once in a lifetime screening.

In Uganda, screening with HPV testing is currently limited to demonstration projects, and thus baseline coverage is low.^36^ The population of Uganda is only 16% urban,^37^ so while a screening program focusing limited financial, laboratory, and human resources on urban centers might screen some of the same women several times between ages 30 and 49 years, such a program is unlikely to achieve high population coverage. Expanding screening coverage to rural areas— where the vast majority of the population resides— will be logistically challenging, but HPV self‐collection facilitated by community health workers may be feasible and may reduce the burden on the limited number of health facilities that provide screening. A recent randomized trial in an impoverished area of Kampala, Uganda that found 99% of women approached for self‐collection of HPV specimens at home or work participated, compared with 48% of women invited to attend the clinic for screening.^12^ Whether this increase in screening uptake occurs in rural settings remains to be seen. The feasibility of expanding coverage to remote areas might be improved if screening can be integrated into existing HIV clinics that serve women with and without HIV infection. A recent survey of HIV networks in sub‐Saharan Africa indicated that on‐site access to cervical cancer screening with HPV testing was available at 18% of surveyed facilities, with cryotherapy available at 57% of sites.^38^ None of the HIV facilities that offer HPV testing in the survey were in Uganda,^38^ although some Ugandan clinics offer VIA and cryotherapy.^39^ Integration of HIV and cervical cancer screening programs has been identified as potentially beneficial by stakeholders in Uganda, if health worker shortages can be addressed.^39^, ^40^ Realizing synergies with HIV networks and investing in community health workers may facilitate increased screening coverage in Uganda, but will require additional investment from countries and international donors.

There are limitations to this analysis. Because we did not have costing data to reflect expansion of screening coverage with self‐collection in a community setting, our estimates of the cost‐effectiveness of expanding coverage are based on provider‐collection at the clinic. However, a previous analysis suggests that differences in test performance and effectiveness between collection methods are small.^14^, ^16^ Thus, if the costs of a screening program relying on HPV self‐collection in a community‐based setting are similar to or less than the costs of a program relying on provider‐collection, the impact on the cost‐effectiveness profile of expanding coverage will likely be small as well. Data on women's time and transportation costs are also limited, but may impact the relative cost‐effectiveness of expanding coverage versus increasing frequency if women living near screening and treatment facilities face shorter travel and wait times than women in remote areas; however, HPV self‐collection may reduce women's travel and time costs if only HPV‐positive women need to attend the clinic.

Our assumption of equivalent loss to follow‐up rates for expanding coverage versus increasing screening frequency may be an oversimplification. For instance, if coverage expansion efforts focus on outreach to rural areas while programmatic increases in screening frequency focus on urban areas where women may already have greater access to care, linkage to treatment may differ between strategies. Gas‐based cryotherapy relies on consistent resupply of gas, which is expensive to transport and not always available. In remote areas, women may need to travel farther to reach a facility with cryotherapy equipment, increasing the risk of loss to follow‐up. However, new ablative technologies currently undergoing testing are smaller, portable, and do not require gas,^9^, ^29^, ^41^ potentially improving linkage to treatment for screen‐positive women in both urban and rural areas.

We did not consider the potential impact of differential risk of disease among women who might benefit from increases in programmatic coverage versus those who might benefit from increased frequency of screening. If, for instance, program efforts focus on providing multiple screening opportunities for HIV‐infected women— who have a greater prevalence of oncogenic HPV^42^, ^43^ and increased incidence of cervical cancer^44^, ^45^— our analysis comparing coverage expansions in the general population of screening‐eligible women does not adequately capture the relevant tradeoffs. Because the microsimulation model was calibrated to reflect age‐specific HPV prevalence and cancer incidence in the general population, findings must be interpreted accordingly, and do not necessarily apply to groups with higher than average risk.

Both the cost‐effectiveness profile and recurrent financial costs must be favorable to implement a sustainable screening program. Here we present information on both the cost‐effectiveness and affordability of screening in Uganda, at varying frequencies and screening coverage levels, to convey both the relative value and the costs of different programs. Our findings suggest that prioritizing greater access to at least one screening over number of screenings per woman may yield greater population health benefits, improve distributional equity, and provide greater value for money when baseline screening coverage is low. As coverage extends to a greater proportion of the target population, increasing the frequency of screening may yield additional health benefits while remaining very cost‐effective. We have quantified this programmatic tradeoff between expanding coverage and increasing frequency in order to inform evidence‐based decision‐making by those formulating screening guidelines and implementing new programs in low‐resource settings. Harnessing scarce resources to prioritize expansion of screening coverage may provide an opportunity to strengthen primary health care systems. For the millions of women in low‐ and middle‐income countries who are past the target age for HPV vaccination, secondary prevention with cervical cancer screening is the only way to prevent a leading cause of cancer death in women. Given very low baseline coverage at present, a policy focus on increasing access to screening appears to be compatible with improving both equity and efficiency.

## Supporting information

Supporting Information

## Footnotes

1 A detailed description of the natural history model and parameterization for the United States population has been previously published [15]. The model was then adapted and parameterized for the populations of India, Nicaragua, and Uganda, as described in a separate publication and accompanying appendix [14]. The appendix accompanying this manuscript reiterates the parameterization process for the Uganda model.
