# Supplementary material for: To expand coverage, or increase frequency: Quantifying the tradeoffs between equity and efficiency facing cervical cancer screening programs in low‐resource settings
Source: Int J Cancer. 2017 Jan 27;140(6):1293–305. doi: 10.1002/ijc.30551 (PMC5516173; doi:10.1002/ijc.30551)
Supplement: Supplementary file 1 — Supporting Information [file IJC-140-1293-s001.docx]

**Appendix:**

**To Expand Coverage, or Increase Frequency: Quantifying the Tradeoffs between Equity and Efficiency Facing Cervical Cancer Screening Programs in Low-Resource Settings**

This appendix provides additional details on methods, assumptions, and results presented in the main manuscript.

**MODEL CALIBRATION**

**Overview of the calibration process**

Details of the model development process, including initial parameterization and calibration, have been previously published but we summarize these here [[1](#_ENREF_1), [2](#_ENREF_2)]. Derivation of model parameter values requires an iterative process involving comprehensive literature reviews, data synthesis and analysis, consultations with experts, and explorations of the influence of uncertain parameters and assumptions in the model. Baseline HPV incidence rates, as a function of genotype and age, were derived from published data from a prospective cohort of sexually active women aged 15-85 years in Bogota, Colombia [[3](#_ENREF_3)]. Because HPV incidence is known to vary by population as a function of sexual behaviors, age-specific HPV incidence and natural immunity following initial infection were considered important candidates for calibration. Transitions occurring from the HPV state (i.e., time-dependent rates of HPV clearance and progression by genotype) were informed by primary longitudinal data from the control arm of the Costa Rica Vaccine Trial [[4](#_ENREF_4)]. Type-specific data on CIN2 and CIN3 regression and progression are limited [[5-10](#_ENREF_5)], so these parameters were also candidates for calibration. Because of the computational intensity of microsimulation models, we selected parameters for calibration based on the availability of 1) a range of plausible values and 2) good empirical data to inform calibration targets (i.e., high-risk HPV prevalence to calibrate HPV incidence rates; cancer incidence to calibrate CIN2 and CIN3 regression and progression rates).

To calibrate the model, we set plausible search ranges around baseline input values for age- and type-specific HPV incidence, as well as natural immunity following initial infection and progression and regression of CIN, and performed repeated model simulations in the absence of any preventive intervention. For each simulation, we randomly selected a single value for each of the uncertain parameters from the identified plausible range, creating a unique vector of parameter values (i.e., parameter “set”). Following over 1,475,000 repeated samplings, we identified the parameter sets with the highest correspondence to the empirical calibration target data by calculating and aggregating the log-likelihood of model-projected outcomes. We used the 50 parameter sets with the highest likelihood scores (i.e., best overall fit to the empirical data) from each country for analysis to capture uncertainty in the model parameters as a form of probabilistic sensitivity analysis. We report the mean outcomes across these top 50 parameter sets; incremental cost-effectiveness ratios are reported as the ratio of the mean costs divided by the mean effects of one strategy versus another across sets.

**Calibration targets**

Model calibration to Uganda has been previously published [[2](#_ENREF_2)]. We assessed model fit by observing projected model outcomes of age-specific prevalence of high-risk HPV and age-specific cancer incidence relative to empirical data. The likelihood-based scoring algorithm for Uganda only included age-specific cancer incidence, as we observed a better fit to cancer incidence data when we did not include HPV prevalence in the scoring algorithm; however, we still considered visual fit to HPV prevalence to arrive at the final scoring algorithm.

Age-specific prevalence of high-risk HPV was drawn from START-UP data on *care*HPV positivity using a cut-off ratio cut-point of 0.5 relative light units (**Tables A.1**). For each age group, we derived a 95% binomial confidence interval around the point prevalence, which comprised the calibration target.

Age-specific cancer incidence was drawn from the Kampala registry in *Cancer in Five Continents* [[11](#_ENREF_11)] (**Tables A.2**). The likelihood function for each age group was assumed to follow a normal distribution.

Composite goodness-of-fit scores for each input parameter set were generated by summing the log likelihood of each model outcome (i.e., age-specific cancer incidence). The 50 input parameter sets with the highest goodness-of-fit scores thus yielded the model outputs that were simultaneously closest to all calibration targets, and were selected for analysis. **Figures A.1** and **A.2** display model fit to epidemiologic data on age-specific prevalence of high-risk HPV and age-specific cancer incidence in Uganda.

**COST DATA**

**Direct Medical Costs: Screening, Diagnosis, and Treatment of Precancerous Lesions**

The direct medical costs of screening, diagnosis, and treatment of precancerous lesions were drawn from the Screening Technologies to Advance Rapid Testing for Cervical Cancer Prevention–Utility and Program Planning (START-UP) demonstration study in Uganda (Kampala) [[2](#_ENREF_2), [12](#_ENREF_12)]. Direct medical costs included clinical staff time, clinical supplies, drugs, clinical equipment, laboratory staff time, laboratory supplies, and laboratory equipment.

We report costs in 2011 international dollars (I$) to facilitate comparisons across regions. The relevant GDP deflators were applied to local currency units to inflate to year 2011 levels, and local currency units were then converted to international dollars by means of purchasing power parity (PPP) exchange rates [[13](#_ENREF_13)]. The exceptions were for equipment, which was generally procured in the United States, and the cost of the careHPV test kit, which was assumed to be US$5. For these tradable goods, one international dollar is equivalent to one U.S. dollar. Costs are reported in **Table 1** of the main manuscript.

**Cost of Cancer Care by Stage**

Costs associated with cancer care by stage (Local vs. Regional or Distant), including direct medical costs, women's time costs for time spent receiving care, women's transportation costs to health facilities, and cancer staging costs were derived from previous analyses and converted to 2011 I$ as described above. Cancer care costs were based on primary data from Kenya, as we have previously described [[14](#_ENREF_14), [15](#_ENREF_15)]. Costs are reported in **Table 1** of the main manuscript.

**Women’s Time and Transportation Costs**

We derived women’s time costs from the United Nations Development Programme Human Development Indicator, “Estimated GNI per capita, female”, which was derived from the ratio of female to male wage, female and male shares of economically active population, and gross national income (GNI) and reported in constant 2011 I$ [[16](#_ENREF_16)]. We assumed this represented annual income for working 40 hours per week, 50 weeks per year to estimate an average hourly wage (**Table 1**).

Estimates for time spent traveling, waiting, and receiving care was dependent upon the facility level where care was assumed to take place (**Table A.3**). Women’s time estimates for round-trip transportation and waiting were obtained from prior studies in Kenya (**Table A.4**) [[14](#_ENREF_14), [17](#_ENREF_17)]. Estimates of women’s time spent receiving a procedure were based on site-specific data from the START-UP demonstration projects, with staff time spent on the procedure (excluding preparation and registration time, which we assumed were built into patient waiting time) used as a proxy for women’s procedure time. Round-trip transportation costs to each health facility level were obtained from previous analyses [[14](#_ENREF_14), [15](#_ENREF_15), [17](#_ENREF_17)] and converted to 2011 I$; these are reported in **Table 1** of the main manuscript.

**PROTOCOLS FOR TREATMENT OF PRECANCER**

We assumed HPV DNA testing was primarily a two-visit strategy, where screening occurred during the first visit and women returned to the clinic to receive results (and treatment with cryotherapy, if screen-positive and eligible) in a second visit. To account for the fact that women in a two-visit strategy have had the opportunity to consider the possibility of treatment prior to receiving results, we assumed 80% would receive same-day cryotherapy, with the remaining women delaying treatment. Women who delayed treatment accrued the time and transportation costs of an additional clinic visit unless they were lost to follow-up.

Women who screened positive who were ineligible for cryotherapy were assumed to be referred to a secondary facility for colposcopy and subsequent treatment. Treatment protocols were based on information from in-country clinicians familiar with standard of care and availability of and preferences for treatment options. We assumed that, upon a histologic diagnosis of CIN1, women received cryotherapy at a secondary facility; a histologic diagnosis of CIN2/3 was followed by cryotherapy for approximately 80% of women, and LEEP for approximately 20% of women, and treatment occurred at a secondary facility.

We assumed cytology required separate visits for screening (primary facility), receiving results (primary facility), colposcopy (secondary facility), and subsequent treatment (secondary facility).

Loss-to-follow-up rates impact cost accrual in the microsimulation model, and we have the flexibility to input differential loss-to-follow-up for each visit (i.e., results, cryotherapy (if delayed), diagnostic confirmation, and treatment following diagnostic confirmation). In the base case, we assumed 10% of women who deferred cryotherapy would ultimately be lost to follow-up. We assumed visits for screening results, diagnostic confirmation, and treatment following diagnostic confirmation were each associated with 15% loss-to-follow-up.

Following treatment of precancerous lesions with either cryotherapy or LEEP, we assumed the setting-specific follow-up protocols as used in the START-UP demonstration study (**Table A.5**). We included direct medical costs of each procedure, as well as women’s time and transportation costs (as shown in **Table 1** of the main manuscript). While women in the START-UP study could be seen prior to scheduled follow-up visits as necessary, we did not have data on these unscheduled visits. Treatment complications in each site were very rare, so we did not consider these costs in the base case analysis.

**EQUATION FOR CALCULATION OF INCREMENTAL NET MONETARY BENEFIT (INMB)[**[**18**](#_ENREF_18)**]**

$$\Delta Life expectancy \times WTP- \Delta Cost$$

where

$\Delta Life expectancy=({Life expectancy}_{improved}$ $- {Life expectancy}_{baseline}$)

$\Delta Cost=({Cost}_{improved}-{Cost}_{baseline}$)

and WTP = willingness-to-pay threshold (i.e., Uganda’s per capita gross domestic product [GDP]

**SUPPLEMENTARY RESULTS**

The incremental cost-effectiveness ratios for screening with HPV testing (provider-collected cervical samples) either once, twice, or three times in a lifetime at each baseline coverage level (30% to 90%), as screening once in a lifetime becomes available at higher coverage levels, are presented in **Tables A.6, A.7, A.8, A.9, A.10, A.11,** and **A.12.**

The health and financial impact, in terms of cases averted per 100,000 women and direct medical cost per 100,000 women, is presented in **Table A.13.**

**Table A.1. Age-specific prevalence of high-risk HPV, Uganda [**[**19**](#_ENREF_19)**].**^a^

| **Age group** | **Number of women** | **Number of women with high-risk HPV** | **Prevalence (95% CI)** |
| --- | --- | --- | --- |
| 25 – 34 years | 1,367 | 426 | 0.31 (0.28, 0.34) |
| 35 – 44 years | 1,131 | 284 | 0.25 (0.23, 0.28) |
| 45 – 54 years | 558 | 127 | 0.22 (0.19, 0.26) |
| 55 – 60 years | 90 | 28 | 0.31 (0.22, 0.42) |

^a^ HPV positivity was based on a cut-off of 0.5 relative light units. We did not include HPV prevalence in our scoring algorithm for Uganda, although we did consider visual fit to HPV prevalence.

**Table A.2. Age-specific cervical cancer incidence, Uganda (Kyadondo registry, 2003-2007)[**[**11**](#_ENREF_11)**].**^a^

| **Age group** | **Cases** | **Rate per 100,000 women (95% CI)** |
| --- | --- | --- |
| 25 – 29 years | 42 | 7.6 (5.3, 9.9) |
| 30 – 34 years | 84 | 26.5 (20.8, 32.2) |
| 35 – 39 years | 111 | 53.7 (43.7, 63.7) |
| 40 – 44 years | 138 | 99.7 (83.1, 116.3) |
| 45 – 49 years | 105 | 121.7 (98.4, 145.0) |
| 50 – 54 years | 108 | 181.3 (147.1, 215.5) |
| 55 – 59 years | 59 | 163.2 (121.6, 204.8) |
| 60 – 64 years | 68 | 199.7 (152.2, 247.2) |
| 65 – 69 years | 33 | 145.8 (96.1, 195.6) |
| 70 – 74 years | 35 | 175.0 (117.0, 233.0) |

^a^ Although our scoring algorithm included cancer incidence in aged 40 years and above, we considered visual fit to all age groups.

**Table A.3. Location of Service Delivery for Screening, Diagnosis, and Treatment of Precancerous Lesions and Cancer.**^a^

| **Procedure** | | **Location of services** |
| --- | --- | --- |
| HPV DNA test | | Primary facility |
| Cytology test | | Primary facility |
| VIA test | | Primary facility |
| Colposcopy/biopsy | | Secondary facility |
| Cryotherapy | | Primary facility (for women eligible for screen-and-treat cryotherapy)  Secondary facility (for women ineligible for screen-and-treat cryotherapy |
| LEEP | | Secondary facility |
| Follow-up visits (after cryotherapy or LEEP) | | Primary facility (for examinations and Pap)  Secondary facility (if colposcopy is necessary) |
| Cancer treatment | | Tertiary facility |
| ^a^ | HPV: human papillomavirus; LEEP: loop electrosurgical excision procedure; VIA: visual inspection with acetic acid. | |

**Table A.4. Women’s Time Spent Receiving Care.**^a^

| **Procedure** | | **Time Spent Receiving Care (Minutes)** | | | |
| --- | --- | --- | --- | --- | --- |
|  | |  |  |  | **Uganda[**[**14**](#_ENREF_14)**,** [**19**](#_ENREF_19)**]** |
| **Screening**^b^ | | | | | |
| Wait time  Procedure time  Transport time (round-trip) | | |  |  | 90 |
|  |  |  |  |  | 15 |
|  |  |  |  |  | 220 |
| Receiving results (negative) 10 | | | | | |
| Receiving results (positive) 15 | | | | | |
| **Diagnosis** | | | | | |
| Wait time | | |  |  | 180 |
| Procedure time  Transport time (round-trip) | | |  |  | 35 |
|  |  |  |  |  | 340 |
|  | | | | | |
| **Treatment of Precancer: Screen-and-Treat Cryotherapy** ^c^ | | | | | |
| Wait time  Procedure time  Transport time (round-trip) | | |  |  | 90 |
|  |  |  |  |  | 30 |
|  |  |  |  |  | 220 |
|  | | | | | |
| **Treatment of Precancer: LEEP** | | | | | |
| Wait time  Procedure time  Transport time (round-trip) | | |  |  | 180 |
|  |  |  |  |  | 25 |
|  |  |  |  |  | 340 |
| ^a^ | I$: international dollars. LEEP: loop electrosurgical excision procedure. | | | | |
| ^b^ | We assumed women received screening in the first visit and results in a second visit. Thus, wait time does not include the laboratory processing time for HPV samples. | | | | |
| ^c^ | We assumed most eligible women received cryotherapy in the same visit they received screening results (i.e., initial screening visit with VIA; second visit for HPV testing). Thus, additional transportation time was only accrued for women who delayed cryotherapy. Screen-and-treat cryotherapy was assumed to take place at a primary facility. For women who received cryotherapy following diagnostic confirmation of CIN, wait time and transport time were the same as for LEEP, as cryotherapy was assumed to take place at a secondary facility. | | | | |

**Table A.5. Follow-up Protocols after Treatment of Precancerous Lesions.**^a^

| **Treatment** | |  |  |  | **Uganda** |
| --- | --- | --- | --- | --- | --- |
| **Cryotherapy** | | | | | |
|  | |  |  |  | 6 week exam |
|  | |  |  |  | 1 year Cytology/Colposcopy^b^ |
|  | | | | | |
| **LEEP** | | | | | |
|  | |  |  |  | 6 week exam |
|  | |  |  |  | 1 year Cytology  1 year Colposcopy, as needed^b^ |
| a | LEEP: loop electrosurgical excision procedure. Follow-up protocols were based on the START-UP demonstration study in each setting. We included direct medical costs and women’s time and transportation costs for each procedure. A 6 week visual exam was associated with the same costs as VIA at the primary facility. Cytology was assumed to take place at a primary facility, while colposcopy was assumed to take place at a secondary facility. | | | | |
| b | Colposcopy at 1 year was performed as needed in Uganda. Approximately 15% of women who received treatment required a colposcopy and biopsy at 1 year for suspected recurrence. | | | | |

**Table A.6. Incremental cost-effectiveness ratios, baseline coverage: 30%.**^a^

| **Strategy** | **Cancer reduction (%)** | **Discounted lifetime costs (I$)** | **Discounted life expectancy (years)** | **ICER (I$/YLS)** |
| --- | --- | --- | --- | --- |
| *Baseline coverage: 30%* | | | | |
| No screening | -- | 12.42 | 25.20221 | -- |
| HPV 1x, 30% cov | 10.9 | 15.92 | 25.22714 | 140 |
| HPV 2x, 30% cov | 17.1 | 18.55 | 25.23708 | 260 |
| HPV 3x, 30% cov | 20.5 | 20.28 | 25.24032 | 540 |
|  |  |  |  |  |
| *If HPV 1x can achieve 40% coverage:* | | | | |
| HPV 1x, 30% cov | 10.9 | 15.92 | 25.22714 | 140 |
| HPV 1x, 40% cov | 14.5 | 17.10 | 25.23526 | 150 |
| HPV 2x, 30% cov | 17.1 | 18.55 | 25.23708 | Dominated |
| HPV 3x, 30% cov | 20.5 | 20.28 | 25.24032 | 630 |
|  |  |  |  |  |
| *If HPV 1x can achieve 50% coverage:* | | | | |
| HPV 1x, 30% cov | 10.9 | 15.92 | 25.22714 | 140 |
| HPV 1x, 50% cov | 18.0 | 18.28 | 25.24328 | 150 |
| HPV 2x, 30% cov | 17.1 | 18.55 | 25.23708 | Dominated |
| HPV 3x, 30% cov | 20.5 | 20.28 | 25.24032 | Dominated |
|  |  |  |  |  |
| *If HPV 1x can achieve 60% coverage:* | | | | |
| HPV 1x, 30% cov | 10.9 | 15.92 | 25.22714 | 140 |
| HPV 1x, 60% cov | 21.6 | 19.45 | 25.25146 | 150 |
| HPV 2x, 30% cov | 17.1 | 18.55 | 25.23708 | Dominated |
| HPV 3x, 30% cov | 20.5 | 20.28 | 25.24032 | Dominated |
|  |  |  |  |  |
| *If HPV 1x can achieve 70% coverage:* | | | | |
| HPV 1x, 30% cov | 10.9 | 15.92 | 25.22714 | 140 |
| HPV 1x, 70% cov | 25.4 | 20.59 | 25.26000 | 140 |
| HPV 2x, 30% cov | 17.1 | 18.55 | 25.23708 | Dominated |
| HPV 3x, 30% cov | 20.5 | 20.28 | 25.24032 | Dominated |
|  |  |  |  |  |
| *If HPV 1x can achieve 80% coverage:* | | | | |
| HPV 1x, 30% cov | 10.9 | 15.92 | 25.22714 | 140 |
| HPV 1x, 80% cov | 29.1 | 21.76 | 25.26831 | 140 |
| HPV 2x, 30% cov | 17.1 | 18.55 | 25.23708 | Dominated |
| HPV 3x, 30% cov | 20.5 | 20.28 | 25.24032 | Dominated |
|  |  |  |  |  |
| *If HPV 1x can achieve 90% coverage:* | | | | |
| HPV 1x, 30% cov | 10.9 | 15.92 | 25.22714 | Dominated |
| HPV 1x, 90% cov | 32.9 | 22.90 | 25.27677 | 140 |
| HPV 2x, 30% cov | 17.1 | 18.55 | 25.23708 | Dominated |
| HPV 3x, 30% cov | 20.5 | 20.28 | 25.24032 | Dominated |

^a^ Cov: proportion of the target population covered by screening; HPV: human papillomavirus DNA testing; I$: 2011 international dollars; 1x: screening once in a lifetime at age 30 years; 2x: screening twice in a lifetime at ages 30 and 40 years; 3x: screening three times in a lifetime at ages 30, 40, and 50 years. For strategies involving screening two or three times in a lifetime, we assumed the same women (based on the screening coverage level) receive all screenings, while remaining women are never screened.

**Table A.7. Incremental cost-effectiveness ratios, baseline coverage: 40%.**^a^

| **Strategy** | **Cancer reduction** | **Discounted lifetime costs (I$)** | **Discounted life expectancy (years)** | **ICER (I$/YLS)** |
| --- | --- | --- | --- | --- |
| *Baseline coverage: 40%* | | | | |
| No screening | -- | 12.42 | 25.20221 | -- |
| HPV 1x, 40% cov | 14.5 | 17.10 | 25.23526 | 140 |
| HPV 2x, 40% cov | 22.8 | 20.61 | 25.24831 | 270 |
| HPV 3x, 40% cov | 27.2 | 22.93 | 25.25271 | 530 |
|  |  |  |  |  |
| *If HPV 1x can achieve 50% coverage:* | | | | |
| HPV 1x, 40% cov | 14.5 | 17.10 | 25.23526 | 140 |
| HPV 1x, 50% cov | 18.0 | 18.28 | 25.24328 | 150 |
| HPV 2x, 40% cov | 22.8 | 20.61 | 25.24831 | 460 |
| HPV 3x, 40% cov | 27.2 | 22.93 | 25.25271 | 530 |
|  |  |  |  |  |
| *If HPV 1x can achieve 60% coverage:* | | | | |
| HPV 1x, 40% cov | 14.5 | 17.10 | 25.23526 | 140 |
| HPV 1x, 60% cov | 21.6 | 19.45 | 25.25146 | 150 |
| HPV 2x, 40% cov | 22.8 | 20.61 | 25.24831 | Dominated |
| HPV 3x, 40% cov | 27.2 | 22.93 | 25.25271 | 2,780 |
|  |  |  |  |  |
| *If HPV 1x can achieve 70% coverage:* | | | | |
| HPV 1x, 40% cov | 14.5 | 17.10 | 25.23526 | Dominated |
| HPV 1x, 70% cov | 25.4 | 20.59 | 25.26000 | 140 |
| HPV 2x, 40% cov | 22.8 | 20.61 | 25.24831 | Dominated |
| HPV 3x, 40% cov | 27.2 | 22.93 | 25.25271 | Dominated |
|  |  |  |  |  |
| *If HPV 1x can achieve 80% coverage:* | | | | |
| HPV 1x, 40% cov | 14.5 | 17.10 | 25.23526 | Dominated |
| HPV 1x, 80% cov | 29.1 | 21.76 | 25.26831 | 140 |
| HPV 2x, 40% cov | 22.8 | 20.61 | 25.24831 | Dominated |
| HPV 3x, 40% cov | 27.2 | 22.93 | 25.25271 | Dominated |
|  |  |  |  |  |
| *If HPV 1x can achieve 90% coverage:* | | | | |
| HPV 1x, 40% cov | 14.5 | 17.10 | 25.23526 | Dominated |
| HPV 1x, 90% cov | 32.9 | 22.90 | 25.27677 | 140 |
| HPV 2x, 40% cov | 22.8 | 20.61 | 25.24831 | Dominated |
| HPV 3x, 40% cov | 27.2 | 22.93 | 25.25271 | Dominated |

^a^ Cov: proportion of the target population covered by screening; HPV: human papillomavirus DNA testing; I$: 2011 international dollars; 1x: screening once in a lifetime at age 30 years; 2x: screening twice in a lifetime at ages 30 and 40 years; 3x: screening three times in a lifetime at ages 30, 40, and 50 years. For strategies involving screening two or three times in a lifetime, we assumed the same women (based on the screening coverage level) receive all screenings, while remaining women are never screened.

**Table A.8. Incremental cost-effectiveness ratios, baseline coverage: 50%.**^a^

| **Strategy** | **Cancer reduction** | **Discounted lifetime costs (I$)** | **Discounted life expectancy (years)** | **ICER (I$/YLS)** |
| --- | --- | --- | --- | --- |
| *Baseline coverage: 50%* | | | | |
| No screening | -- | 12.42 | 25.20221 | -- |
| HPV 1x, 50% cov | 18.0 | 18.28 | 25.24328 | 140 |
| HPV 2x, 50% cov | 28.4 | 22.67 | 25.25946 | 270 |
| HPV 3x, 50% cov | 33.9 | 25.56 | 25.26494 | 530 |
|  |  |  |  |  |
| *If HPV 1x can achieve 60% coverage:* | | | | |
| HPV 1x, 50% cov | 18.0 | 18.28 | 25.24328 | 140 |
| HPV 1x, 60% cov | 21.6 | 19.45 | 25.25146 | 140 |
| HPV 2x, 50% cov | 28.4 | 22.67 | 25.25946 | 400 |
| HPV 3x, 50% cov | 33.9 | 25.56 | 25.26494 | 530 |
|  |  |  |  |  |
| *If HPV 1x can achieve 70% coverage:* | | | | |
| HPV 1x, 50% cov | 18.0 | 18.28 | 25.24328 | Dominated |
| HPV 1x, 70% cov | 25.4 | 20.59 | 25.26000 | 140 |
| HPV 2x, 50% cov | 28.4 | 22.67 | 25.25946 | Dominated |
| HPV 3x, 50% cov | 33.9 | 25.56 | 25.26494 | 1,010 |
|  |  |  |  |  |
| *If HPV 1x can achieve 80% coverage:* | | | | |
| HPV 1x, 50% cov | 18.0 | 18.28 | 25.24328 | Dominated |
| HPV 1x, 80% cov | 29.1 | 21.76 | 25.26831 | 140 |
| HPV 2x, 50% cov | 28.4 | 22.67 | 25.25946 | Dominated |
| HPV 3x, 50% cov | 33.9 | 25.56 | 25.26494 | Dominated |
|  |  |  |  |  |
| *If HPV 1x can achieve 90% coverage:* | | | | |
| HPV 1x, 50% cov | 18.0 | 18.28 | 25.24328 | Dominated |
| HPV 1x, 90% cov | 32.9 | 22.90 | 25.27677 | 140 |
| HPV 2x, 50% cov | 28.4 | 22.67 | 25.25946 | Dominated |
| HPV 3x, 50% cov | 33.9 | 25.56 | 25.26494 | Dominated |

^a^ Cov: proportion of the target population covered by screening; HPV: human papillomavirus DNA testing; I$: 2011 international dollars; 1x: screening once in a lifetime at age 30 years; 2x: screening twice in a lifetime at ages 30 and 40 years; 3x: screening three times in a lifetime at ages 30, 40, and 50 years. For strategies involving screening two or three times in a lifetime, we assumed the same women (based on the screening coverage level) receive all screenings, while remaining women are never screened.

**Table A.9. Incremental cost-effectiveness ratios, baseline coverage: 60%.**^a^

| **Strategy** | **Cancer reduction** | **Discounted lifetime costs (I$)** | **Discounted life expectancy (years)** | **ICER (I$/YLS)** |
| --- | --- | --- | --- | --- |
| *Baseline coverage: 60%* | | | | |
| No screening | -- | 12.42 | 25.20221 | -- |
| HPV 1x, 60% cov | 21.6 | 19.45 | 25.25146 | 140 |
| HPV 2x, 60% cov | 33.9 | 24.73 | 25.27076 | 270 |
| HPV 3x, 60% cov | 40.6 | 28.20 | 25.27728 | 530 |
|  |  |  |  |  |
| *If HPV 1x can achieve 70% coverage:* | | | | |
| HPV 1x, 60% cov | 21.6 | 19.45 | 25.25146 | Dominated |
| HPV 1x, 70% cov | 25.4 | 20.59 | 25.26000 | 140 |
| HPV 2x, 60% cov | 33.9 | 24.73 | 25.27076 | 380 |
| HPV 3x, 60% cov | 40.6 | 28.20 | 25.27728 | 530 |
|  |  |  |  |  |
| *If HPV 1x can achieve 80% coverage:* | | | | |
| HPV 1x, 60% cov | 21.6 | 19.45 | 25.25146 | Dominated |
| HPV 1x, 80% cov | 29.1 | 21.76 | 25.26831 | 140 |
| HPV 2x, 60% cov | 33.9 | 24.73 | 25.27076 | Dominated |
| HPV 3x, 60% cov | 40.6 | 28.20 | 25.27728 | 720 |
|  |  |  |  |  |
| *If HPV 1x can achieve 90% coverage:* | | | | |
| HPV 1x, 60% cov | 21.6 | 19.45 | 25.25146 | Dominated |
| HPV 1x, 90% cov | 32.9 | 22.90 | 25.27677 | 140 |
| HPV 2x, 60% cov | 33.9 | 24.73 | 25.27076 | Dominated |
| HPV 3x, 60% cov | 40.6 | 28.20 | 25.27728 | 10,440 |

^a^ Cov: proportion of the target population covered by screening; HPV: human papillomavirus DNA testing; I$: 2011 international dollars; 1x: screening once in a lifetime at age 30 years; 2x: screening twice in a lifetime at ages 30 and 40 years; 3x: screening three times in a lifetime at ages 30, 40, and 50 years. For strategies involving screening two or three times in a lifetime, we assumed the same women (based on the screening coverage level) receive all screenings, while remaining women are never screened.

**Table A.10. Incremental cost-effectiveness ratios, baseline coverage: 70%.**^a^

| **Strategy** | **Cancer reduction** | **Discounted lifetime costs (I$)** | **Discounted life expectancy (years)** | **ICER (I$/YLS)** |
| --- | --- | --- | --- | --- |
| *Baseline coverage: 70%* | | | | |
| No screening | -- | 12.42 | 25.20221 | -- |
| HPV 1x, 70% cov | 25.4 | 20.59 | 25.26000 | 140 |
| HPV 2x, 70% cov | 39.9 | 26.74 | 25.28265 | 270 |
| HPV 3x, 70% cov | 47.7 | 30.79 | 25.29020 | 540 |
|  |  |  |  |  |
| *If HPV 1x can achieve 80% coverage:* | | | | |
| HPV 1x, 70% cov | 25.4 | 20.59 | 25.26000 | Dominated |
| HPV 1x, 80% cov | 29.1 | 21.76 | 25.26831 | 140 |
| HPV 2x, 70% cov | 39.9 | 26.74 | 25.28265 | 350 |
| HPV 3x, 70% cov | 47.7 | 30.79 | 25.29020 | 540 |
|  |  |  |  |  |
| *If HPV 1x can achieve 90% coverage:* | | | | |
| HPV 1x, 70% cov | 25.4 | 20.59 | 25.26000 | Dominated |
| HPV 1x, 90% cov | 32.9 | 22.90 | 25.27677 | 140 |
| HPV 2x, 70% cov | 39.9 | 26.74 | 25.28265 | Dominated |
| HPV 3x, 70% cov | 47.7 | 30.79 | 25.29020 | 590 |

^a^ Cov: proportion of the target population covered by screening; HPV: human papillomavirus DNA testing; I$: 2011 international dollars; 1x: screening once in a lifetime at age 30 years; 2x: screening twice in a lifetime at ages 30 and 40 years; 3x: screening three times in a lifetime at ages 30, 40, and 50 years. For strategies involving screening two or three times in a lifetime, we assumed the same women (based on the screening coverage level) receive all screenings, while remaining women are never screened.

**Table A.11. Incremental cost-effectiveness ratios, baseline coverage: 80%.**^a^

| **Strategy** | **Cancer reduction** | **Discounted lifetime costs (I$)** | **Discounted life expectancy (years)** | **ICER (I$/YLS)** |
| --- | --- | --- | --- | --- |
| *Baseline coverage: 80%* | | | | |
| No screening | -- | 12.42 | 25.20221 | -- |
| HPV 1x, 80% cov | 29.1 | 21.76 | 25.26831 | 140 |
| HPV 2x, 80% cov | 45.5 | 28.80 | 25.29419 | 270 |
| HPV 3x, 80% cov | 54.5 | 33.42 | 25.30289 | 530 |
|  |  |  |  |  |
| *If HPV 1x can achieve 90% coverage:* | | | | |
| HPV 1x, 80% cov | 29.1 | 21.76 | 25.26831 | Dominated |
| HPV 1x, 90% cov | 32.9 | 22.90 | 25.27677 | 140 |
| HPV 2x, 80% cov | 45.5 | 28.80 | 25.29419 | 340 |
| HPV 3x, 80% cov | 54.5 | 33.42 | 25.30289 | 530 |

^a^ Cov: proportion of the target population covered by screening; HPV: human papillomavirus DNA testing; I$: 2011 international dollars; 1x: screening once in a lifetime at age 30 years; 2x: screening twice in a lifetime at ages 30 and 40 years; 3x: screening three times in a lifetime at ages 30, 40, and 50 years. For strategies involving screening two or three times in a lifetime, we assumed the same women (based on the screening coverage level) receive all screenings, while remaining women are never screened.

**Table A.12. Incremental cost-effectiveness ratios, baseline coverage: 90%.**^a^

| **Strategy** | **Cancer reduction** | **Discounted lifetime costs (I$)** | **Discounted life expectancy (years)** | **ICER (I$/YLS)** |
| --- | --- | --- | --- | --- |
| *Baseline coverage: 90%* | | | | |
| No screening | -- | 12.42 | 25.20221 | -- |
| HPV 1x, 90% cov | 32.9 | 22.90 | 25.27677 | 140 |
| HPV 2x, 90% cov | 51.2 | 30.82 | 25.30578 | 270 |
| HPV 3x, 90% cov | 61.3 | 36.02 | 25.31556 | 530 |

^a^ Cov: proportion of the target population covered by screening; HPV: human papillomavirus DNA testing; I$: 2011 international dollars; 1x: screening once in a lifetime at age 30 years; 2x: screening twice in a lifetime at ages 30 and 40 years; 3x: screening three times in a lifetime at ages 30, 40, and 50 years. For strategies involving screening two or three times in a lifetime, we assumed the same women (based on the screening coverage level) receive all screenings, while remaining women are never screened.

**Table A.13. Sensitivity analysis: Incremental cost-effectiveness ratios, at loss to follow-up levels of 40%^a^: baseline coverage 30%, 50%, and 70%.^b^**

| **Strategy** | **Cancer reduction (%)** | **Discounted lifetime costs (I$)** | **Discounted life expectancy (years)** | **ICER (I$/YLS)** |
| --- | --- | --- | --- | --- |
| ***Baseline coverage: 30%*** | | | | |
| No screening | -- | 12.42 | 25.20221 | -- |
| HPV 1x, 30% cov | 6.8 | 15.63 | 25.21731 | 210 |
| HPV 2x, 30% cov | 11.9 | 17.85 | 25.22564 | 270 |
| HPV 3x, 30% cov | 14.8 | 19.32 | 25.22867 | 480 |
|  |  |  |  |  |
| *If HPV 1x can achieve 40% coverage:* | | | | |
| HPV 1x, 30% cov | 6.8 | 15.63 | 25.21731 | 210 |
| HPV 1x, 40% cov | 9.1 | 16.71 | 25.22233 | 210 |
| HPV 2x, 30% cov | 11.9 | 17.85 | 25.22564 | 350 |
| HPV 3x, 30% cov | 14.8 | 19.32 | 25.22867 | 480 |
|  |  |  |  |  |
| *If HPV 1x can achieve 50% coverage:* | | | | |
| HPV 1x, 30% cov | 6.8 | 15.63 | 25.21731 | 210 |
| HPV 1x, 50% cov | 11.4 | 17.78 | 25.22736 | 210 |
| HPV 2x, 30% cov | 11.9 | 17.85 | 25.22564 | Dominated |
| HPV 3x, 30% cov | 14.8 | 19.32 | 25.22867 | 1,170 |
|  |  |  |  |  |
| *If HPV 1x can achieve 60% coverage:* | | | | |
| HPV 1x, 30% cov | 6.8 | 15.63 | 25.21731 | 210 |
| HPV 2x, 30% cov | 11.9 | 17.85 | 25.22564 | Dominated |
| HPV 1x, 60% cov | 13.7 | 18.85 | 25.23240 | 210 |
| HPV 3x, 30% cov | 14.8 | 19.32 | 25.22867 | Dominated |
|  |  |  |  |  |
| *If HPV 1x can achieve 70% coverage:* | | | | |
| HPV 1x, 30% cov | 6.8 | 15.63 | 25.21731 | Dominated |
| HPV 2x, 30% cov | 11.9 | 17.85 | 25.22564 | Dominated |
| HPV 3x, 30% cov | 14.8 | 19.32 | 25.22867 | Dominated |
| HPV 1x, 70% cov | 16.2 | 19.90 | 25.23782 | 210 |
|  |  |  |  |  |
| ***Baseline coverage: 50%*** | | | | |
| *If HPV 1x can achieve 50% coverage:* | | | | |
| HPV 1x, 50% cov | 11.4 | 17.78 | 25.22736 | 210 |
| HPV 2x, 50% cov | 19.8 | 21.49 | 25.24080 | 280 |
| HPV 3x, 50% cov | 24.6 | 23.93 | 25.24588 | 480 |
|  |  |  |  |  |
| *If HPV 1x can achieve 60% coverage:* | | | | |
| HPV 1x, 50% cov | 11.4 | 17.78 | 25.22736 | Dominated |
| HPV 1x, 60% cov | 13.7 | 18.85 | 25.23240 | 210 |
| HPV 2x, 50% cov | 19.8 | 21.49 | 25.24080 | 310 |
| HPV 3x, 50% cov | 24.6 | 23.93 | 25.24588 | 480 |
|  |  |  |  |  |
| *If HPV 1x can achieve 70% coverage:* | | | | |
| HPV 1x, 50% cov | 11.4 | 17.78 | 25.22736 | Dominated |
| HPV 1x, 70% cov | 16.2 | 19.90 | 25.23782 | 210 |
| HPV 2x, 50% cov | 19.8 | 21.49 | 25.24080 | Dominated |
| HPV 3x, 50% cov | 24.6 | 23.93 | 25.24588 | 500 |
|  |  |  |  |  |
| *If HPV 1x can achieve 80% coverage:* | | | | |
| HPV 1x, 50% cov | 11.4 | 17.78 | 25.22736 | Dominated |
| HPV 1x, 80% cov | 18.6 | 20.97 | 25.24296 | 210 |
| HPV 2x, 50% cov | 19.8 | 21.49 | 25.24080 | Dominated |
| HPV 3x, 50% cov | 24.6 | 23.93 | 25.24588 | 1,020 |
|  |  |  |  |  |
| *If HPV 1x can achieve 90% coverage:* | | | | |
| HPV 1x, 50% cov | 11.4 | 17.78 | 25.22736 | Dominated |
| HPV 2x, 50% cov | 19.8 | 21.49 | 25.24080 | Dominated |
| HPV 1x, 90% cov | 20.9 | 22.02 | 25.24833 | 210 |
| HPV 3x, 50% cov | 24.6 | 23.93 | 25.24588 | Dominated |
|  |  |  |  |  |
| ***Baseline coverage: 70%*** | | | | |
| *If HPV 1x can achieve 70% coverage:* | | | | |
| HPV 1x, 70% cov | 16.2 | 19.90 | 25.23782 | 210 |
| HPV 2x, 70% cov | 27.8 | 25.11 | 25.25641 | 280 |
| HPV 3x, 70% cov | 34.6 | 28.53 | 25.26347 | 480 |
|  |  |  |  |  |
| *If HPV 1x can achieve 80% coverage:* | | | | |
| HPV 1x, 70% cov | 16.2 | 19.90 | 25.23782 | Dominated |
| HPV 1x, 80% cov | 18.6 | 20.97 | 25.24296 | 210 |
| HPV 2x, 70% cov | 27.8 | 25.11 | 25.25641 | 310 |
| HPV 3x, 70% cov | 34.6 | 28.53 | 25.26347 | 480 |
|  |  |  |  |  |
| *If HPV 1x can achieve 90% coverage:* | | | | |
| HPV 1x, 70% cov | 16.2 | 19.90 | 25.23782 | Dominated |
| HPV 1x, 90% cov | 20.9 | 22.02 | 25.24833 | 210 |
| HPV 2x, 70% cov | 27.8 | 25.11 | 25.25641 | 380 |
| HPV 3x, 70% cov | 34.6 | 28.53 | 25.26347 | 480 |

^a^ Loss to follow-up is defined as the proportion of women who do not return for each subsequent clinical encounter, relative to the previous visit. Loss to follow-up applies to the results visit following *care*HPV testing, the cryotherapy visit (only for the 20% of women who receive positive screening test results but do not receive immediate cryotherapy in the same visit), and the diagnostic confirmation visit and treatment visit for women who are ineligible for cryotherapy.

^b^ Cov: proportion of the target population covered by screening; HPV: human papillomavirus DNA testing; I$: 2011 international dollars; 1x: screening once in a lifetime at age 30 years; 2x: screening twice in a lifetime at ages 30 and 40 years; 3x: screening three times in a lifetime at ages 30, 40, and 50 years. For strategies involving screening two or three times in a lifetime, we assumed the same women (based on the screening coverage level) receive all screenings, while remaining women are never screened.

**Table A.14. Health impact and affordability, by screening frequency and coverage level.**^a^

| **Strategy** | **Cases averted per 100,000 women** | **Cost per 100,000 women (US$)**^b^ |
| --- | --- | --- |
| HPV 1x, 30% cov | 410 | 240,000 |
| HPV 1x, 40% cov | 548 | 320,000 |
| HPV 1x, 50% cov | 681 | 400,000 |
| HPV 1x, 60% cov | 817 | 480,000 |
| HPV 1x, 70% cov | 962 | 570,000 |
| HPV 1x, 80% cov | 1,101 | 650,000 |
| HPV 1x, 90% cov | 1,242 | 730,000 |
|  |  |  |
| HPV 2x, 30% cov | 648 | 460,000 |
| HPV 3x, 30% cov | 774 | 640,000 |
|  |  |  |
| HPV 2x, 40% cov | 862 | 610,000 |
| HPV 3x, 40% cov | 1,030 | 850,000 |
|  |  |  |
| HPV 2x, 50% cov | 1,073 | 760,000 |
| HPV 3x, 50% cov | 1,283 | 1,070,000 |
|  |  |  |
| HPV 2x, 60% cov | 1,281 | 910,000 |
| HPV 3x, 60% cov | 1,534 | 1,280,000 |
|  |  |  |
| HPV 2x, 70% cov | 1,507 | 1,070,000 |
| HPV 3x, 70% cov | 1,805 | 1,490,000 |
|  |  |  |
| HPV 2x, 80% cov | 1,720 | 1,220,000 |
| HPV 3x, 80% cov | 2,062 | 1,710,000 |
|  |  |  |
| HPV 2x, 90% cov | 1,935 | 1,370,000 |
| HPV 3x, 90% cov | 2,319 | 1,920,000 |

^a^ Cov: proportion of the target population covered by screening; HPV: human papillomavirus DNA testing; US$: 2013 United States dollars; 1x: screening once in a lifetime at age 30 years; 2x: screening twice in a lifetime at ages 30 and 40 years; 3x: screening three times in a lifetime at ages 30, 40, and 50 years. For strategies involving screening two or three times in a lifetime, we assumed the same women (based on the screening coverage level) receive all screenings, while remaining women are never screened.

^b^ Costs include undiscounted direct medical costs only; women’s time and transportation costs are not included. Cost projections include screening and management of screen-positive women (i.e., further diagnostic testing and treatment of cancer), but do not include the cost of treating detected cancer.

**Figure A.1.** Selected model output from the top 50 input parameter sets compared with empirical data (i.e., calibration targets) on age-specific prevalence of high-risk HPV in Uganda, based on a relative light unit cut-off value of 0.5 in the START-UP studies [[19](#_ENREF_19)]. Bold lines represent the 95% confidence intervals around the empirical data, and gray circles represent model output from each of the top 50 input parameter sets.

**Figure A.2.** Selected model output from the top 50 input parameter sets compared with empirical data (i.e., calibration targets) on age-specific cancer incidence in Uganda (Kyadondo registry, 2003-2007)[[11](#_ENREF_11)]. Bold lines represent the 95% confidence intervals around the empirical data, and gray circles represent model output from each of the top 50 input parameter sets.

**References**

1. Campos NG, Burger EA, Sy S, et al. An updated natural history model of cervical cancer: derivation of model parameters*.* *Am J Epidemiol* 2014;180(5):545-555.

2. Campos NG, Tsu, V., Jeronimo, J., Mvundura, M., Lee, K., Kim, J.J. When and how often to screen for cervical cancer in three low- and middle-income countries: A cost-effectiveness analysis*.* *Papillomavirus Research* 2015.

3. Munoz N, Mendez F, Posso H, et al. Incidence, duration, and determinants of cervical human papillomavirus infection in a cohort of Colombian women with normal cytological results*.* *J Infect Dis* 2004;190(12):2077-2087.

4. Herrero R, Hildesheim A, Rodriguez AC, et al. Rationale and design of a community-based double-blind randomized clinical trial of an HPV 16 and 18 vaccine in Guanacaste, Costa Rica*.* *Vaccine* 2008;26(37):4795-4808.

5. McCredie MR, Sharples KJ, Paul C, et al. Natural history of cervical neoplasia and risk of invasive cancer in women with cervical intraepithelial neoplasia 3: a retrospective cohort study*.* *Lancet Oncol* 2008;9(5):425-434.

6. Meyskens FL, Jr., Surwit E, Moon TE, et al. Enhancement of regression of cervical intraepithelial neoplasia II (moderate dysplasia) with topically applied all-trans-retinoic acid: a randomized trial*.* *J Natl Cancer Inst* 1994;86(7):539-543.

7. Keefe KA, Schell MJ, Brewer C, et al. A randomized, double blind, Phase III trial using oral beta-carotene supplementation for women with high-grade cervical intraepithelial neoplasia*.* *Cancer Epidemiol Biomarkers Prev* 2001;10(10):1029-1035.

8. Castle PE, Schiffman M, Wheeler CM, et al. Evidence for frequent regression of cervical intraepithelial neoplasia-grade 2*.* *Obstet Gynecol* 2009;113(1):18-25.

9. Wang SM, Colombara D, Shi JF, et al. Six-year regression and progression of cervical lesions of different human papillomavirus viral loads in varied histological diagnoses*.* *Int J Gynecol Cancer* 2013;23(4):716-723.

10. Moscicki AB, Ma Y, Wibbelsman C, et al. Rate of and risks for regression of cervical intraepithelial neoplasia 2 in adolescents and young women*.* *Obstet Gynecol* 2010;116(6):1373-1380.

11. Forman D, Bray, F., Brewster, D.H., Gombe Mbalawa, C., Kohler, B., Piñeros, M., Steliarova-Foucher, E., Swaminathan, R., Ferlay, J. (eds) *Cancer Incidence in Five Continents, Vol. X*, 2013, IARC: Lyon, France.

12. Mvundura M and Tsu V. Estimating the costs of cervical cancer screening in high-burden Sub-Saharan African countries*.* *Int J Gynaecol Obstet* 2014;126(2):151-155.

13. *World Development Indicators*, 2016, World Bank.

14. Goldie SJ, Gaffikin L, Goldhaber-Fiebert JD, et al. Cost-effectiveness of cervical-cancer screening in five developing countries*.* *N Engl J Med* 2005;353(20):2158-2168.

15. Campos NG, Kim JJ, Castle PE, et al. Health and economic impact of HPV 16/18 vaccination and cervical cancer screening in Eastern Africa*.* *Int J Cancer* 2012;130(11):2672-2684.

16. United Nations Development Programme. *International Human Development Indicators*. 2014; Available from: <http://hdr.undp.org/en/data>.

17. Campos NGM, M.; Alfaro, K.; Gage, J.C.; Castle, P.E.; Felix, J.; Cremer, M.L.; Kim, J.J. The Comparative and Cost-Effectiveness of HPV-Based Cervical Cancer Screening in El Salvador. *Int J Cancer* 2015;137:893-902.

18. Kim JJ, Campos NG, Sy S, et al. Inefficiencies and High-Value Improvements in U.S. Cervical Cancer Screening Practice: A Cost-Effectiveness Analysis*.* *Ann Intern Med* 2015;163(8):589-597.

19. Jeronimo J, Bansil P, Lim J, et al. A multicountry evaluation of careHPV testing, visual inspection with acetic acid, and papanicolaou testing for the detection of cervical cancer*.* *Int J Gynecol Cancer* 2014;24(3):576-585.
